# Determination of ethanol, isopropyl alcohol and methanol in alcohol-based hand sanitiser to ensure product quality, safety and efficacy

**DOI:** 10.1038/s41598-023-36283-1

**Published:** 2023-06-10

**Authors:** Nur Dayana Nisbar, Sara Khalida Jamal Khair, Nur Baizura Bujang, Ahmad Yusri Mohd Yusop

**Affiliations:** Centre of Compliance and Quality Control, National Pharmaceutical Regulatory Agency, Ministry of Health Malaysia, Selangor, Malaysia

**Keywords:** Chemical biology, Chemistry

## Abstract

The Coronavirus Disease-2019 (COVID-19) outbreak is an unprecedented global pandemic, sparking grave public health emergencies. One of the measures to reduce COVID-19 transmissions recommended by the World Health Organization is hand hygiene, i.e., washing hands with soap and water or disinfecting them using an alcohol-based hand sanitiser (ABHS). Unfortunately, competing ABHSs with unknown quality, safety, and efficacy thrived, posing yet another risk to consumers. This study aims to develop, optimise, and validate a gas chromatography-mass spectrometry (GC–MS)-based analytical method to simultaneously identify and quantify ethanol or isopropyl alcohol as the active ingredient in ABHS, with simultaneous determination of methanol as an impurity. The GC–MS was operated in Electron Ionisation mode, and Selected Ion Monitoring was chosen as the data acquisition method for quantitation. The analytical method was validated for liquid and gel ABHSs, covering the specificity, linearity and range, accuracy, and precisions, including the limit of detection and the limit of quantitation. The specificity of each target analyte was established using the optimised chromatographic separation with unique quantifier and qualifier ions. The linearity was ascertained with a coefficient of determination (r^2^) of > 0.9994 over the corresponding specification range. Respectively, the accuracy and precisions were satisfactory within 98.99 to 101.09% and < 3.04% of the relative standard deviation. The method was successfully applied to 69 ABHS samples, where 14 contained insufficient amounts of the active ingredient. Alarmingly, four samples comprised a high amount of methanol ranging from 5.3 to 19.4% with respect to the active alcohol percentage, which may pose significant short- and long-term health issues, leading to life-threatening crises for consumers. The method established would benefit in protecting the public against the potential harm due to substandard or unsafe ABHS products, primarily due to the presence of hazardous impurities such as methanol.

## Introduction

A respiratory illness outbreak in Wuhan, China, led to the discovery of a new infectious disease named Coronavirus Disease-2019 (COVID-19), caused by the severe acute respiratory syndrome coronavirus 2 (SARS-CoV-2). Since then, COVID-19 has advanced into an unprecedented global pandemic that sparked grave public health emergencies. Preventive measures, therefore, remained inevitable to control the spread of infection. Accordingly, public health agencies across the globe recommended hand hygiene as one of the measures to reduce COVID-19 transmissions^1^.

Hand hygiene has been proven to stop outbreaks, reduce transmission of antimicrobial-resistant organisms, and reduce overall infection rates in healthcare facilities^2^. Hence, in 2002, the United States (US) Centers for Disease Control and Prevention revised their hand hygiene guideline in healthcare settings by recommending that healthcare personnel use alcohol-based hand rubs alongside traditional handwashing with soap and water for patient care^3^. The World Health Organisation (WHO) subsequently published a guideline on hand hygiene, which also promotes the use of alcohol-based hand sanitiser (ABHS) among healthcare workers^4^. ABHSs are recommended for non-visible soiled hands by applying an adequate amount of sanitisers to the palm of one hand, successively rubbing both hands, whilst covering all surfaces of the hands and fingers until they are dry^5^.

In a study published in the Emerging Infectious Diseases, Swiss and German researchers found that the original and modified ABHS formulations recommended by the WHO effectively kill the SARS-CoV-2 with a virus reduction factor of ≥ 3.8 and ≥ 5.9, respectively. The study also found that ethanol and isopropyl alcohol (IPA) were efficient in inactivating the virus in 30 s at a concentration of > 30% v/v^6^. The WHO recommends two formulas for ABHS, which differ only in their alcohol constituent, and is at present widely accepted throughout the world. These formulations are Formulation 1 with 80% v/v of ethanol and Formulation 2 with 75% v/v of IPA, in addition to other ingredients such as glycerol, hydrogen peroxide, and sterile/distilled water. No other active or inactive ingredients should be added, which may impact the quality and potency of the product^7^. Generally, ABHS containing 60 to 95% v/v of alcohol kills microorganisms most effectively, provided that at least 2.4 mL of hand sanitisers were applied for 25 to 30 s^1^.

The combination of healthcare experts’ recommendations for ABHS and consumers’ fear of contracting the novel influenza A (H1N1) swine flu have contributed to the earliest significant spike in hand sanitiser sales in 2009^8^. During the current COVID-19 pandemic, again, ABHS proved to be a crucial tool to reduce the number of SARS-CoV-2 transmissions via skin-to-skin contact. At the early stage of the COVID-19 outbreak, a sudden surge in demand for hand sanitisation products has led to shortages in their supply. Hand sanitiser sales increased by 470% in the first week of March 2020 compared to the same week the previous year, according to Nielsen market research in the US^8^. As a result, many regulatory bodies have relaxed their regulations requirements in response to the significant shortages of hand sanitisers^8,9^.

Recognising the opportunity and at the same time keeping up with consumers’ demands, cosmetic and skincare brands have also diversified their product lines to include ABHS^10^. While these companies often have the technology and ingredients to manufacture ABHS, there are concerns that these new manufacturers are not aware of their regulatory obligations. Although health regulatory authorities have guided the industry to produce ABHS during a public health emergency, some manufacturers may have resorted to in-house formulations that are not validated and licenced for use^11^.

The US Food and Drug Administration (FDA) has recently uncovered grave safety concerns with some hand sanitisers during their quality testing in July 2020. These include low levels of active ingredients, including the detection of hazardous amounts of impurities such as methanol. In addition, some of these products were even labelled with false, misleading, or unproven claims. Consequently, more than 150 hand sanitisers have been recalled and are no longer advised for use^12^. Based on these findings, health regulatory authorities were alerted about substandard or unsafe ABHS products that may become toxic to human health and the environment when used.

In Malaysia, ABHSs are classified as cosmetics or generic products depending on their intended use. Since the COVID-19 pandemic, various manufacturers and companies have filed notification applications with the National Pharmaceutical Regulatory Agency (NPRA) to import, manufacture, or market ABHS as cosmetics. Generally, cosmetic products must be notified, adhere to the requirements set in the Guidelines for Control of Cosmetic Products in Malaysia, and be manufactured by a premise that meets the requirements of Good Manufacturing Practice for cosmetic products or equivalent^13^. In addition, the NPRA has set a specific requirement for ABHS, stating that the active ingredient must contain at least 60% of alcohol and the manufacturer can only make hygiene and antibacterial claims^14^. Notified cosmetic products in Malaysia are monitored from time to time, with regulatory penalties issued upon products that did not comply with the requirements set by the NPRA, including cancellation of notification and removal from the market.

Alcoholmeter, hydrometer, or other chemical analyses with equivalent or greater accuracy are among the most common methods used for determining alcohol concentration^15,16^. During the COVID-19 public health emergency, the National Institute of Standards and Technology (NIST) has supported the industry by developing and evaluating analytical methods for their applicability in identifying and quantifying ethanol and other impurities in 72 hand sanitisers^17^. These methods include gas chromatography with flame ionisation detection (GC-FID), liquid chromatography with ultraviolet absorbance detection (LC-UV), quantitative nuclear magnetic resonance (q-NMR) spectroscopy, and attenuated total reflectance Fourier-transform infrared (ATR-FTIR) spectroscopy.

The NPRA, as the regulatory body in charge of cosmetic product compliance and quality control in Malaysia, herein reported the development of a gas chromatography-mass spectrometry (GC–MS)-based analytical method to simultaneously identify and quantify ethanol, IPA, and methanol in ABHSs. The method was carefully optimised and validated, ensuring robust and reliable performance for determining alcohols and related impurity in liquid and gel ABHS products. The applicability of this method was demonstrated using 69 samples obtained from the Malaysian market under the NPRA’s post-registration market surveillance programme.

## Methods

### Materials

All reagents and solvents were of LC grade and purchased from Merck KGaA (Darmstadt, Germany). The purity of ethanol, IPA, methanol, 2-butanol, and acetonitrile were ≥ 99.90%, ≥ 99.90%, ≥ 99.97%, ≥ 99.00%, and ≥ 99.90%, respectively, as stated on the solvents’ specification.

### Instrumentation and analytical method

The analysis was carried out using a GC system coupled to a quadrupole MS (GC–MS TQ8040, Shimadzu Corporation, Japan). The compounds were separated using a BP-624 GC capillary column (Part number: 054840, Trajan, Australia) with dimensions of 30 m (length) × 0.25 mm (internal diameter) × 1.4 µm (film thickness). Helium was used as the carrier gas at a flow rate of 1 mL/min. The split injection mode was selected, using a split ratio of 1:100 with an injection volume of 0.2 µL. The GC oven temperature was programmed using an initial temperature of 60 °C with a hold time of 3.00 min, then increased to 90 °C at 30 °C/min, and subsequently to 230 °C at 55 °C/min. The final temperature was held for 3.00 min with a total run time of 9.55 min. The injection and interface temperatures were set at 230 °C.

The MS was operated at 70 eV in Electron Ionisation (EI) mode, while the ion source temperature was set at 200 °C. Target analytes were identified by collecting the full-scan mass spectra at an *m/z* range of 25 to 200. The identity of these analytes in a sample was confirmed using the NIST 14 mass spectral library. Selected Ion Monitoring (SIM) mode was chosen as the data acquisition method for quantitation. One target ion was selected as a quantifier ion for each analyte, while two reference ions were chosen for identification purposes. Table 1 shows the quantifier and qualifier ions for each target analyte and internal standard (IS). All data were processed using Lab Solution Software (GC–MS Solution version 2.50, Shimadzu Corporation, Japan).

**Table 1:** The quantifier and qualifier ions for target analytes, including the internal standard.

Target analyte	Quantifier ion (*m/z*)	Qualifier ions (*m/z*)
Methanol	31	Qualifier ion 1: 32 Qualifier ion 2: 29
Ethanol	45	Qualifier ion 1: 46 Qualifier ion 2: 29
Isopropyl alcohol	43	Qualifier ion 1: 41 Qualifier ion 2: 59
2-Butanol (Internal standard)	59	Qualifier ion 1: 41 Qualifier ion 2: 31

### Standard preparation

Standard stock solutions of labelled alcohols (ethanol and IPA), methanol, and 2-butanol (IS) were prepared at concentrations of 5.0% v/v, 0.5% v/v, and 10.0% v/v, respectively, by dissolving their neat standards in acetonitrile. For ethanol and IPA, the calibration solutions were prepared at concentrations of 0.45, 0.55, 0.65, 0.75, 0.85, and 0.95% v/v by diluting the standard stock solution with an appropriate amount of acetonitrile. Calibration solutions for methanol were prepared by aliquoting the standard stock solution in acetonitrile to obtain final concentrations of 0.0115, 0.0175, 0.0275, 0.0375, 0.0450, and 0.0550% v/v. Each ethanol, IPA, and methanol calibration solution was then added with an exact amount of 2-butanol as an IS to yield a final concentration of 0.55% v/v.

### Sample preparation

The developed method was applied to 69 ABHS products obtained from the Malaysian market in liquid and gel forms; collected under the NPRA’s post-registration market surveillance programme. These products were selected based on their over-claim advertisements, public complaints, manufacturer’s track record and random market sampling. These samples were initially homogenised before analysis. For liquid-type hand sanitisers, 0.2 mL of a sample was accurately pipetted into a 20 mL volumetric flask. For gel-type hand sanitisers, their specific gravity at 25 °C (weight/mL) was initially determined using a density meter (Mettler Toledo, Greifensee, Switzerland). Based on the specific gravity, an amount equivalent to 0.2 mL of a gel sample was weighed in a 20 mL volumetric flask. Next, an exact amount of IS stock solution was added to obtain a final concentration of 0.55% v/v. The sample was further diluted to volume with acetonitrile and subsequently vortexed for 2 min until well mixed. The sample solution was finally filtered using a 0.45 µm nylon filter into a GC vial for analysis.

### Method validation

The method was validated according to the International Council for Harmonisation (ICH) Harmonized Tripartite Guideline: Validation of Analytical Procedures: Text and Methodology Q2(R1)^18^. The validation parameters for determining the two labelled alcohols (ethanol and IPA) in liquid and gel ABHSs include specificity, linearity and range, accuracy, and precisions. Methanol, in contrast, is regarded as an impurity in hand sanitiser products, and thus, two additional validation parameters, i.e., limit of detection (LOD) and limit of quantitation (LOQ), were performed. Blank matrices of liquid and gel ABHSs, free from any analyte of interests, were used for method optimisation and validation.

The specificity was evaluated by establishing the ability of the method to discriminate each target analyte in the presence of various interferences originating from other analytes, IS, diluent, and sample matrices. The linearity was established using a six-point calibration curve, analysed in triplicate. The calibration curve was constructed by plotting the peak area ratio of each target analyte versus the IS against their corresponding concentration in % v/v. The linearity range of each target analyte was selected according to their respective specification or limit. The accuracy was established using a recovery study of samples spiked at three quality control levels (low, medium, high) covering the range of the analytical method. The precisions were determined by evaluating the repeatability and intermediate precision. The repeatability was ascertained by analysing six different liquid and gel samples spiked at a single concentration (0.75% v/v ethanol and IPA, and 0.0375% v/v methanol). The intermediate precision was determined using a similar procedure to that of repeatability, albeit the analysis was performed by a second analyst and on a different day. The LOD and LOQ of methanol were initially calculated based on the signal-to-noise (S/N) ratio approach. Based on these estimates, the LOD was then determined experimentally at the lowest concentration of methanol that can be reliably detected, with at least a 3:1 S/N ratio. At the same time, the LOQ was set at a concentration where methanol can be quantified with acceptable accuracy and precisions, giving an S/N ratio of no less than 10.

## Results

### Method development and optimisation

The simultaneous separation of multiple analytes can be a challenging task. Therefore, careful consideration must be given to each target analyte to achieve an excellent chromatographic separation for reliable identification and quantitation. A non-polar (5%-phenyl)-methylpolysiloxane, HP-5 ms (30 m × 0.25 mm × 0.25 µm) column was initially assessed during the early stage of method development, with an initial column temperature of 40 °C. Although a relatively good separation (resolution factor of 1.933 to 4.993) was observed between each target analyte, the peak shape was poor, with tailing factors in the range of 0.96 to 2.359. Moreover, the target analytes’ capacity factors (K’) were observed between 0.059 and 0.320, indicating poorly retained alcohols in the HP-5 ms column. Therefore, another column, i.e., an intermediate polar (6%-cyanopropylphenyl)-dimethylpolysiloxane, BP-624 (30 m × 0.25 mm × 1.4 µm), was evaluated in a review of the first column. Contrarily to the HP-5 ms column, the BP-624 column's thicker stationary phase film of 1.4 µm necessitated a higher initial column temperature of 60 °C. The BP-624 column significantly improved the peak shape of all target analytes. The peaks of all three alcohols were sharp and symmetrical with tailing factors between 1.032 and 1.206. Furthermore, target analytes retention in the column improved significantly, with K’ ranging from 0.219 to 1.107. The BP-624 column was chosen as it provides an optimal chromatographic separation.

The injection volume was evaluated at 0.2, 0.3, and 0.5 µL. The detector response for ethanol and IPA was observed to be saturated, notably at concentrations higher than 0.75% v/v using injection volumes of 0.3 and 0.5 µL. Conversely, the detector saturation for ethanol and IPA was not visible at the injection volume of 0.2 µL. The detector response for methanol, however, was not significantly reduced, with a good response recorded at the lowest injection volume. Based on these findings, an optimum injection volume of 0.2 µL was selected for the final method.

### Method validation

#### Specificity

**Figure 1:**
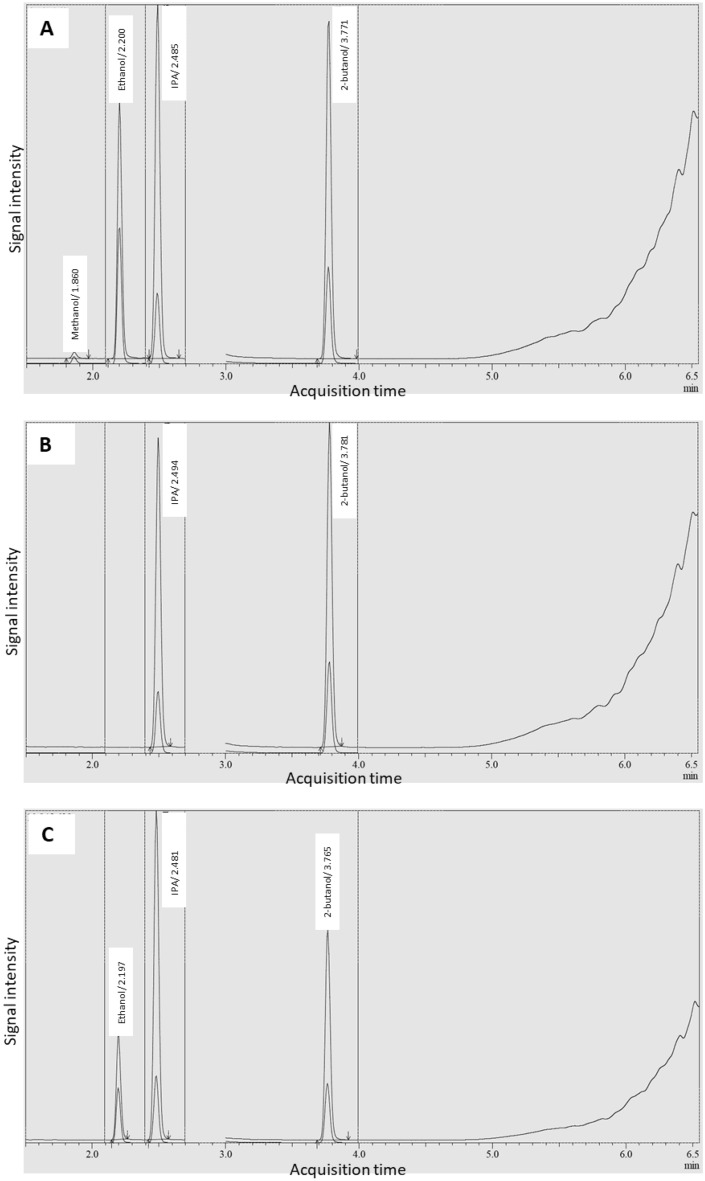
A representative GC-MS total ion chromatograms (TIC) with the addition of 2-butanol as an internal standard. TIC of mixed standard solution (**A**), unspiked sample solution (**B**) and spiked sample solution (**C**), demonstrating the specificity of the method.

All target analytes were chromatographically resolved from one another; identified by their specific retention time, as shown in Fig. 1A, and selectively resolved using the MS SIM detection with unique quantifier and qualifier ions, presented in Table 1. No interferences were found at the peaks of methanol, ethanol, and IPA, as evidenced in the GC–MS total ion chromatograms of an acetonitrile blank solution, an IS of 2-butanol, and a sample solution spiked with IS. Furthermore, the retention times of each standard in the unspiked and spiked samples correspond to their respective peaks in the chromatograms of the mixed standard solution, as shown in Figs. 1B and 1C, respectively. In addition, the reference ions ratio of an unspiked sample and a spiked sample was within the maximum-permitted tolerance of ± 10%, set according to the reference ions ratio of a mixed standard solution.

#### Linearity and range

A linear regression model was applied to the standard curves, and the linearity was determined based on the coefficient of determination (r^2^). The linearity model was further verified using the lack-of-fit test to confirm that the selected regression analysis is accurate at a 95% confidence interval. The r^2^ obtained using six standard concentration levels for ethanol, IPA, and methanol were 0.9994, 0.9995, and 0.9996, respectively. Ethanol and IPA standards were prepared using a range of 4.5 to 9.5% v/v, which is equivalent to 45 to 95% v/v in real samples. Methanol, in contrast, was prepared within a range of 0.01128 to 0.055% v/v, corresponding to 1.128 to 5.5% v/v in real samples. The linear regression model demonstrated excellent linearity between the peak area ratio of the target analytes with respect to 2-butanol (IS) versus their concentration. In addition, all y-intercept values passed zero, established via the student t-test.

#### Accuracy

Results obtained from the spike recovery studies are presented in Table 2. For liquid hand sanitiser samples, the mean recoveries of ethanol, IPA, and methanol were 100.31%, 101.09%, and 99.98%, respectively. While for gel hand sanitiser samples, the mean recoveries were 99.26% for ethanol, 98.99% for IPA, and 99.79% for methanol.

**Table 2:** The accuracy of ethanol, isopropyl alcohol, and methanol at low, medium, and high-quality control levels for liquid and gel hand sanitisers (established via spike recovery studies).

Form	Analyte	Spike concentration (% v/v)	Mean recovery (%) ± 95% CI	RSD (%)	n
Liquid	Ethanol	0.480	101.2 ± 0.004	0.011	6
		0.750	100.2 ± 0.009	0.015	6
		0.900	99.5 ± 0.017	0.023	6
	IPA	0.480	100.5 ± 0.003	0.008	6
		0.750	100.2 ± 0.005	0.008	6
		0.900	99.5 ± 0.006	0.009	6
	Methanol	0.0120	98.5 ± 1.292	1.640	6
		0.0375	99.9 ± 0.687	0.859	6
		0.0500	101.5 ± 0.741	0.912	6
Gel	Ethanol	0.480	97.7 ± 0.004	0.011	6
		0.750	101.0 ± 0.003	0.006	6
		0.900	99.1 ± 0.008	0.011	6
	IPA	0.480	97.9 ± 0.002	0.005	6
		0.750	99.8 ± 0.003	0.005	6
		0.900	99.3 ± 0.006	0.009	6
	Methanol	0.0120	97.3 ± 0.624	0.801	6
		0.0375	101.8 ± 0.435	0.534	6
		0.0500	100.5 ± 1.077	1.340	6

#### Repeatability

The repeatability data are shown in Table 3. The relative standard deviation (RSD) percentages of ethanol, IPA, and methanol spiked in liquid hand sanitiser samples were 1.50%, 0.82%, and 0.83%, respectively. The RSD percentages for gel hand sanitiser samples were 0.57% for ethanol, 0.47% for IPA, and 0.57% for methanol. Overall, each target analyte demonstrated acceptable repeatability with RSD percentages of < 5%.

**Table 3:** The repeatability of ethanol, isopropyl alcohol, and methanol for liquid and gel hand sanitisers.

Form	Analyte	Spike concentration (% v/v)	Mean observed concentration (% v/v) ± 95% CI	RSD (%)	n
Liquid	Ethanol	0.750	0.752 ± 0.0090	1.500	6
	IPA	0.750	0.764 ± 0.0050	0.823	6
	Methanol	0.0375	0.0375 ± 0.0003	0.859	6
Gel	Ethanol	0.750	0.757 ± 0.0035	0.570	6
	IPA	0.750	0.749 ± 0.0028	0.474	6
	Methanol	0.0375	0.0382 ± 0.0002	0.534	6

#### Intermediate precision

The intermediate precision results are shown in Table 4. The RSD percentages for ethanol, IPA, and methanol spiked in liquid hand sanitiser samples were calculated at 1.11%, 3.04%, and 0.81%, respectively. The gel hand sanitiser samples yielded RSD percentages of 0.95% for ethanol, 0.91% for IPA, and 1.56% for methanol. All target analytes demonstrated acceptable intermediate precisions with RSD percentages of < 5%.

**Table 4:** The intermediate precision of ethanol, isopropyl alcohol, and methanol for liquid and gel hand sanitisers.

Form	Analyte	Spike concentration (% v/v)	Mean observed concentration (% v/v) ± 95% CI	RSD (%)	n
Liquid	Ethanol	0.750	0.749 ± 0.0037	0.611	6
	IPA	0.750	0.725 ± 0.0112	1.923	6
	Methanol	0.0375	0.0373 ± 0.0003	0.838	6
Gel	Ethanol	0.750	0.745 ± 0.0022	0.362	6
	IPA	0.750	0.752 ± 0.0074	1.226	6
	Methanol	0.0375	0.0373 ± 0.0004	1.387	6

#### Limit of detection and limit of quantitation

The S/N for methanol via the predetermined LOD and LOQ levels were 517.97 and 1447.44, respectively. Upon confirmation, the RSD percentages of the peak area ratio for LOD (n = 3) was 1.003%, while LOQ (n = 10) was 2.053%. Accordingly, the LOD for methanol was established experimentally at 0.004% v/v (equivalent to 0.4% v/v in a sample); whereas the LOQ was verified at 0.0115% v/v or 1.15% v/v of methanol in a sample.

## Discussion

GC, in general, provides information on the retention time, verifying the identity of alcohols and, simultaneously, quantifying their concentration, making it useful for the quality control of ABHS products. In contrast to the recommended method in the US Pharmacopeia (USP) General Chapter 611, Alcohol Determination^19^, a GC coupled to an MS detector was utilised in this study instead of an FID.

An FID is recommended for alcohol determination because it is easy to use, generating a consistent and adequate response within the specified concentration range, even after dilutions during the sample preparation process. Hence, most analytical instrument companies have published GC-FID application notes as a tool for hand sanitiser analysis^20–22^. Nonetheless, an MS is more sensitive and capable of detecting trace amounts of impurities^23^. Thus, a GC–MS-based method was selected to evaluate ABHS products formulated with ethanol or IPA as the labelled active ingredient.

The method described in this study was developed following the updated US FDA guideline and their subsequent published method^12^, which requires the active ingredient of either ethanol or IPA to be tested for the presence of methanol (as an impurity) if it is obtained from a third-party source. This reference method, however, was established to screen potentially harmful impurities with interim limits, which differs from the guideline published by the NPRA. For instance, under the US FDA temporary policies, ethanol used to manufacture hand sanitiser products containing less than 630 ppm of methanol is not considered contaminated; and is not subjected to adulteration charges under the Food, Drug, and Cosmetic Act. Conversely, the NPRA focused on detecting methanol as an impurity because it is listed in the NPRA Cosmetic Guideline ‘Annex III-Part 1-List of substances which cosmetic products must not contain except subject to restrictions and conditions laid down’^24^. This annexe outlines that methanol content in cosmetic products should not exceed 5%, calculated as a percentage of either ethanol or IPA. If any manufacturer fails to follow this limit, the ABHS notification will be revoked, and their product will be withdrawn from the market.

Numerous global reports have revealed that ABHSs may contain undeclared methanol^25,26^. Therefore, NPRA has been actively analysing ABHSs for compliance with the regulations and legislation. Methanol is toxic when inhaled, taken orally, or applied to the skin; and should never be incorporated into any hand hygiene product. Methanol poisoning may lead to central nervous system depression and severe metabolic acidosis, causing blindness, confusion, and eventual death^27^. Generally, methanol poisoning is rare; typically occurs due to accidental ingestion or suicidal intent. Unfortunately, during the COVID-19 pandemic, there was a proliferation of fatal methanol poisoning resulting from the inappropriate use of hand sanitisers illegally containing methanol^28,29^.

Between August 2020 and November 2021, 69 distinct ABHSs were analysed by the NPRA, with the results presented in Table 5. A discrepancy between the label claim and the analysis result regarding the type of alcohol was evident in one sample. Sample SL18 was labelled with 80.0% ethanol but was instead detected to contain 66.4% IPA. According to the US FDA, IPA should be in the range of 70.0 to 91.3% v/v in any hand sanitiser product^9^. Ethanol, on the contrary, should be between 60.0 and 95.0% v/v^9^. This finding leads to another grave concern regarding the potency of alcohol in the ABHSs. Of the 69 samples tested, 14 were less potent as they contained insufficient amounts of the active ingredient. For instance, five samples were quantified below 60% for ethanol, while nine were quantified below 70% for IPA. Hand sanitisers with a lower alcohol percentage may not work as well for many germs and may merely inhibit the growth of germs rather than killing them altogether.

**Table 5:** The identification and quantitation of labelled alcohols (ethanol and isopropyl alcohol) and their impurity (methanol) in liquid and gel hand sanitiser samples.

No	Sample dosage form	Sample code	Type of alcohol claimed	Amount of EtOH/IPA claimed (% v/v)	Type of alcohol found	Amount of alcohol found (% v/v)	Amount of MeOH (% v/v)	Amount of MeOH (% to active alcohol)
1	Liquid	SL1	IPA	70.0	IPA	70.0	–	–
2		SL2	NA	NA	ND	ND	17.0	–
3		SL3	EtOH	75.0	EtOH	74.2	4.5	6.1
4		SL4	IPA	70.0	IPA	70.3	–	–
5		SL5	IPA	70.0	IPA	62.6	12.1	19.4
6		SL6	EtOH	83.0	EtOH	75.0	–	–
7		SL7	IPA	75.0	IPA	80.0	–	–
8		SL8	EtOH	75.0	EtOH	61.2	1.2	1.9
9		SL9	NA	NA	ND	ND	–	–
10		SL10	EtOH	75.0	EtOH	81.1	–	–
11		SL11	EtOH	78.1	EtOH	74.6	–	–
12		SL12	EtOH	75.0	EtOH	74.8	–	–
13		SL13	EtOH	75.0	EtOH	77.0	–	–
14		SL14	IPA	70.0	IPA	73.4	–	–
15		SL15	IPA	75.0	IPA	74.5	–	–
16		SL16	EtOH	75.0	EtOH	83.3	–	–
17		SL17	EtOH	6.7%	EtOH	< 45.0	–	–
18		SL18	EtOH	80.0	IPA	66.4	–	–
19		SL19	EtOH	70.0	EtOH	67.5	–	–
20		SL20	IPA	70.0	IPA	68.5	–	–
21		SL21	IPA	75.5	IPA	72.0	–	–
22		SL22	IPA	75.0	IPA	77.0	–	–
23		SL23	EtOH	75.0	EtOH	77.0	–	–
24		SL24	IPA	72.0	IPA	80.0	–	–
25	Gel	SG1	EtOH	70.0	EtOH	76.9	–	–
26		SG2	EtOH	66.0	EtOH	71.3	–	–
27		SG3	IPA	70.0	IPA	71.9	–	–
28		SG4	IPA	75.0	IPA	64.1	–	–
29		SG5	IPA	70.0	IPA	57.7	1.7	3.0
30		SG6	IPA	70.0	IPA	74.3	–	–
31		SG6	EtOH	62.0	EtOH	64.9	–	–
32		SG7	EtOH	62.0	EtOH	57.0	–	–
33		SG8	EtOH	75.0	EtOH	67.6	< LOQ	NA
34		SG9	EtOH	68.0	EtOH	69.2	–	–
35		SG10	EtOH	70.0	EtOH	< 45.0	–	–
36		SG11	IPA	70.0	IPA	57.1	–	–
37		SG12	EtOH	75.0	EtOH	75.4	–	–
38		SG13	EtOH	70.0	EtOH	46.4	–	–
39		SG14	EtOH	75.0	EtOH	61.3	3.2	5.3
40		SG15	IPA	75.0	IPA	< 45.0	–	–
41		SG16	EtOH	75.0	EtOH	53.0	–	–
42		SG17	EtOH	80.0	ETOH	70.3	3.9	5.5
43		SG18	EtOH	62.0	EtOH	60.5	–	–
44		SG19	EtOH	70.0	EtOH	69.9	–	–
45		SG20	EtOH	60.0—71.5	EtOH	61.7	–	–
46		SG21	EtOH	60.0—71.5	EtOH	60.0	–	–
47		SG22	EtOH	68.0	EtOH	65.0	–	–
48		SG23	EtOH	70.0	EtOH	65.3	–	–
49		SG24	EtOH	76.0	EtOH	72.4	–	–
50		SG25	IPA	75.0	IPA	74.6	–	–
51		SG26	EtOH	75.0	EtOH	69.5	–	–
52		SG27	EtOH	66.0	EtOH	72.3	–	–
53		SG28	EtOH	62.0	EtOH	63.5	–	–
54		SG29	EtOH	75.0	EtOH	67.0	–	–
55		SG30	EtOH	NA	EtOH	63.0	–	–
56		SG31	EtOH	NA	EtOH	73.0	–	–
57		SG32	EtOH	68.0	EtOH	60.0	–	–
58		SG33	EtOH	68.0	EtOH	60.0	–	–
59		SG34	EtOH	75.0	EtOH	77.0	–	–
60		SG35	EtOH	65.0	EtOH	72.0	–	–
61		SG36	EtOH	70.0	EtOH	63.8	–	–
62		SG37	EtOH	72.9	EtOH	72.0	–	–
63		SG38	IPA	70.0	IPA	52.0	–	–
64		SG39	EtOH	75.0	EtOH	61.9	–	–
65		SG40	EtOH	63.0	EtOH	68.4	–	–
66		SG41	EtOH	70.0	EtOH	71.3	–	–
67		SG42	EtOH	75.0	EtOH	73.1	–	–
68		SG43	EtOH	66.4	EtOH	65.1	–	–
69		SG44	IPA	NA	IPA	56.3	–	–

*EtOH* ethanol, *IPA* isopropyl alcohol, *MeOH* methanol, *NA* not applicable, *ND* not detected, *LOQ* limit of quantitation.

Alarmingly, four samples, viz. samples SL3, SL5, SG14, and SG17, may pose a significant threat to consumers as they contained a high amount of methanol ranging from 5.3 to 19.4% with respect to the active alcohol percentage. Therefore, consumers who have been exposed to ABHSs containing methanol should discontinue using them immediately and seek medical attention if they experience any concerning symptoms. NPRA, at the same time, will cancel the products’ notification and remove these products from the market to protect consumers’ health and well-being.

## Conclusion

A GC–MS-based method was developed, optimised, and validated for the quality, safety, and efficacy assessment of ABHS products. The method can be utilised to identify and quantify ethanol or IPA as the active ingredient in ABHS, with simultaneous determination of methanol, an impurity that should never be present in any hand sanitiser products. The method's applicability was demonstrated using 69 ABHS samples obtained in liquid and gel forms from the Malaysian market. The strategies discussed herein would be beneficial to protect the public against the potential harm of substandard or unsafe ABHS products. Therefore, it is critical for a regulatory body like the NPRA to ensure that ABHSs contain the required percentage of alcohol and are free of any potentially hazardous impurities like methanol.

## Data Availability

The datasets generated during and/or analysed during the current study are not publicly available to protect some sensitive information in the raw data from being accessed by others outside the regulatory agencies but are available from the corresponding author on reasonable request.
